# Supplementary material for: Digital transition in rural emergency medicine: Impact of job satisfaction and workload on communication and technology acceptance
Source: PLoS One. 2023 Jan 24;18(1):e0280956. doi: 10.1371/journal.pone.0280956 (PMC9873191; doi:10.1371/journal.pone.0280956)
Supplement: S1 Appendix — Questions concerning knowledge transfer and knowledge sources. (DOCX) [file pone.0280956.s001.docx]

**Appendix 1: Questionnaire**

**Questions concerning knowledge transfer and knowledge sources**

1. The implementation of new work equipment, new software, etc. is usually done through personal exchange and observation at the workplace. [specialist, explicit, individual]
2. To enhance development, there are regular employee interviews or corresponding reports or questionnaires in our organization. [specialist, explicit, organizational]
3. In our organization, cross-team or cross-group meetings are held regularly in order to improve cooperation among each other. [social, explicit, organizational]
4. Compliance with procedural instructions and process procedures is comprehensively reviewed and continuously improved in our organization (e.g. documentation). [method, explicit, organizational]
5. The procedures in our organization (e.g. emergency call acceptance, shock room handover, vehicle disinfection) are clearly defined and known to every employee as much as necessary. [method, explicit, individual]
6. When making important decisions, each employee can make his or her own arguments and is involved in the decision-making process. [social, implicit, individual]
7. The employees of our organization also engage in joint activities outside of working hours (e.g. football, cinema, bowling). [social, implicit, organizational]
8. In our organization, even short-term teams and shift groups coordinate quickly and work together productively and smoothly. [method, implicit, organizational]
9. If something is difficult to explain in words but has to be demonstrated (e.g. operation of new devices), there is always a colleague who takes over. [specialist, implicit, individual]
10. In our organization, employees make sure that everyone does their job in accordance with the rules and, if necessary, directs attention to colleagues for misconduct. [method, implicit, individual]
11. In our team / area we know each other very well and know how to use and compensate for our strengths and weaknesses in such a way that we complement each other ideally. [specialist, implicit, organizational]
12. In our team /area it is clearly regulated who is responsible for specific topics and a person to contact for everyone else. [social, explicit, individual]
13. In our organization there are sufficient documents available on questions of everyday work as well as relevant specialist literature (e.g., SOP, specialist journals). [source internal, document]
14. Work results, process changes, etc. are documented (e.g., therapy success rate, conversion to new software applications). [source internal, document]
15. Our organization is supported by external experts (e.g., employees of medical device manufacturers, software providers). [source external, person]
16. There is a regular exchange of information with other organizations in the rescue or treatment chain, which are part of the patient care before or after treatment in our organization (e.g., dispatcher center, ambulances, hospital). [source external, organization]
17. There are internal quality circles on internal innovations and idea management, which serve for continuous improvement and quality assurance in our organization. [source internal, organization]
18. Our organization cooperates successfully with external institutions (e.g., universities, institutes) in research and development of treatments, operational strategies, etc. [source external, organization]
19. Our organization regularly uses external training (e.g., conferences, congresses, seminars) in the region. [source external, person]
20. Public research institutions or competent institutions regularly provide our organization with practical research or study results. [source external, document]
21. New employees receive advice and induction support for their job in our organization. [source internal, person]
22. Higher-level institutions (e.g., special purpose associations) actively support our organization in technical development and problem solving. [source internal, organization]
23. Our organization offers internal seminars, workshops, etc., which are led by internal experts of the company. [source internal, person]
24. Our organization offers internal seminars, workshops, etc., which are led by external experts from other companies or institutions. [source external, person]
25. There are sufficient offers for the exchange of knowledge between organizations that have common interests (e.g., interdisciplinary training, case conferences). [source external, organization]
26. Our organization regularly receives information from external companies or institutions (e.g., medical device manufacturers) about their products and services and is also supported by them in their professional development. [source external, document]
27. There is a collection of internal Standard Operating Procedures (SOPs) or procedural instructions for specific operations and situations in our organization. [instrument passive general]
28. In the available documents (digital or analog) I quickly find exactly the information I was looking for. [instrument passive accuracy]
29. Documents are available to all employees and do not represent the opinions of individuals but show generally valid and accepted practices or uniform standards of our organization. [instrument passive availability]
30. All documents are clearly and transparently stored, so that I can quickly decide which of the available documents will help me with a particular problem or task. [instrument passive transparency]
31. At my workplace I always can access the corresponding documents, because there are enough folders (analog) or PCs, etc. (digital). [instrument passive accessibility]
32. The relevant documents are always easy to find, and you can access them quickly and easily. [instrument passive accessibility]
33. The available documents are always up-to-date and will be immediately adapted to changing conditions. [instrument passive actuality]
34. The information or instructions contained here help me systematically and precisely to work on a specific problem or task. [instrument passive demand-oriented]
35. I decide for myself when I call up appropriate specifications and implement them. [instrument passive use-oriented]
36. By using the SOP and procedural instructions, my own competence for processing various tasks improves considerably. [instrument passive competence-oriented]
37. There are external networks or working groups in our organization's regional environment from which you can receive or join information (e.g. regional working groups, thematic project groups, specialist societies). [instrument active general]
38. In information offers, I quickly find exactly the information I was looking for. [instrument active accuracy]
39. Information providers use the skills of all members to advance the common theme, regardless of the sending employer. [instrument active availability]
40. In such information offers, it becomes clear to me very quickly who could provide me with what information or who has specific skills to help me with my own problems or tasks. [instrument active transparency]
41. If I would like to contribute to an offer of information myself, I believe that this is possible in an uncomplicated and fast way, as there are sufficient possibilities. [instrument active accessibility]
42. From my point of view, participation in information offers, i.e., accession, co-author, etc., always happens uncomplicatedly and without much delay. [instrument active accessibility]
43. Information offers always deal with current topics and developments and adapt their orientation to them continuously. [instrument active actuality]
44. Information and contacts from appropriate information offers help me to find solutions to problems and tasks of my work. [instrument active demand-oriented]
45. I decide for myself whether and to what extent I will act with appropriate information offers. [instrument use demand-oriented]
46. Participation in information services significantly improves my skills for solving problems and tasks in everyday work. [instrument active competence-oriented]
