# Supplementary material for: Digital transition in rural emergency medicine: Impact of job satisfaction and workload on communication and technology acceptance
Source: PLoS One. 2023 Jan 24;18(1):e0280956. doi: 10.1371/journal.pone.0280956 (PMC9873191; doi:10.1371/journal.pone.0280956)
Supplement: S2 Appendix — (DOCX) [file pone.0280956.s002.docx]

**Appendix 2**

**Factor structure of the scale for active knowledge transfer
(preliminary and final version)**

|  | Preliminary version | | Final version | |
| --- | --- | --- | --- | --- |
|  | 1 | 2 | 1 | 2 |
| The training in the use of new work equipment, new software, etc. usually takes place through personal exchange and observation at the workplace. | -.030 | .907 |  |  |
| In our organization, cross-team or cross-professional meetings take place regularly in order to improve cooperation with each other. | .763 | .118 | .550 | .515 |
| When making important decisions, each employee can contribute their own arguments and is involved in the decision-making process. | .632 | .287 | .472 | .519 |
| In our organization, even teams and shift groups assembled at short notice coordinate quickly and then work together productively and smoothly. | .598 | .142 | .785 | .112 |
| There are internal quality circles for internal innovations and idea management, which serve in our organization for continuous improvement and quality assurance. | .681 | .207 | .610 | .450 |
| New employees receive advice and induction support for their job in our organization. | .694 | .077 | .710 | .278 |
| There are sufficient offers for the exchange of knowledge between organizations that have common interests (e.g., interdisciplinary trainings, case conferences). | .780 | .087 | .543 | .522 |
| In our organization, internal seminars, workshops, etc. are offered, which are led by internal experts. | .713 | -.047 | .578 | .277 |
| At my workplace, I can always access the corresponding documents, as there are enough folders (analog) or PCs, etc. (digital). | .455 | .516 | .401 | .597 |
| If you can explain something badly with words, but have to demonstrate it (e.g., operation of new devices), there is always a colleague who takes care of it. | .166 | 1.36 | .739 | .083 |
| From my point of view, participation in information supply, i.e., joining, co-authoring, etc., is always uncomplicated and without much delay. |  |  | .067 | .865 |
| Participation in information events significantly improves my skills for solving problems and tasks in everyday work. |  |  | .165 | .774 |
