# Supplementary material for: Digital transition in rural emergency medicine: Impact of job satisfaction and workload on communication and technology acceptance
Source: PLoS One. 2023 Jan 24;18(1):e0280956. doi: 10.1371/journal.pone.0280956 (PMC9873191; doi:10.1371/journal.pone.0280956)
Supplement: S3 Appendix — (DOCX) [file pone.0280956.s003.docx]

**Appendix 3**

Means (and standard deviation) of technology acceptance and knowledge transfer as a function of point in time (pre, post), satisfaction (low, high) and workload (low, high)

| Time | Satisfaction | Workload | Mean | Std. Dev. | n |
| --- | --- | --- | --- | --- | --- |
| *Technoloy acceptance* | |  |  |  |  |
| Phase: Pre | Satisfaction (high) | Workload (low) | 2.75 | 0.78 | 51 |
|  |  | Workload (high) | 2.59 | 0.78 | 27 |
|  |  | Total | 2.70 | 0.78 | 78 |
|  | Satisfaction (low) | Workload (low) | 2.65 | 0.90 | 17 |
|  |  | Workload (high) | 2.44 | 0.79 | 104 |
|  |  | Total | 2.47 | 0.80 | 121 |
|  | Total | Workload (low) | 2.73 | 0.80 | 68 |
|  |  | Workload (high) | 2.47 | 0.79 | 131 |
|  |  | Total | 2.56 | 0.80 | 199 |
| Phase: Post | Satisfaction (high) | Workload (low) | 2.53 | 0.68 | 30 |
|  |  | Workload (high) | 2.22 | 0.86 | 21 |
|  |  | Total | 2.41 | 0.77 | 51 |
|  | Satisfaction (low) | Workload (low) | 2.52 | 0.60 | 9 |
|  |  | Workload (high) | 2.08 | 0.87 | 58 |
|  |  | Total | 2.14 | 0.85 | 67 |
|  | Total | Workload (low) | 2.53 | 0.66 | 39 |
|  |  | Workload (high) | 2.12 | 0.86 | 79 |
|  |  | Total | 2.26 | 0.82 | 118 |
| Total | Satisfaction (high) | Workload (low) | 2.67 | 0.75 | 81 |
|  |  | Workload (high) | 2.43 | 0.83 | 48 |
|  |  | Total | 2.58 | 0.78 | 129 |
|  | Satisfaction (low) | Workload (low) | 2.60 | 0.80 | 26 |
|  |  | Workload (high) | 2.31 | 0.83 | 162 |
|  |  | Total | 2.35 | 0.83 | 188 |
|  | Total | Workload (low) | 2.66 | 0.76 | 107 |
|  |  | Workload (high) | 2.34 | 0.83 | 210 |
|  |  | Total | 2.45 | 0.82 | 317 |
| *Knowledge Transfer* | |  |  |  |  |
| Pre | Satisfaction (high) | Workload (low) | 3.52 | 0.86 | 51 |
|  |  | Workload (high) | 3.48 | 0.95 | 27 |
|  |  | Total | 3.51 | 0.88 | 78 |
|  | Satisfaction (low) | Workload (low) | 2.73 | 0.89 | 17 |
|  |  | Workload (high) | 2.94 | 0.73 | 104 |
|  |  | Total | 2.91 | 0.75 | 121 |
|  | Total | Workload (low) | 3.32 | 0.93 | 68 |
|  |  | Workload (high) | 3.05 | 0.80 | 131 |
|  |  | Total | 3.14 | 0.86 | 199 |
| Post | Satisfaction (high) | Workload (low) | 3.64 | 0.95 | 30 |
|  |  | Workload (high) | 3.67 | 1.13 | 21 |
|  |  | Total | 3.65 | 1.02 | 51 |
|  | Satisfaction (low) | Workload (low) | 3.26 | 0.65 | 9 |
|  |  | Workload (high) | 2.88 | 0.76 | 58 |
|  |  | Total | 2.93 | 0.75 | 67 |
|  | Total | Workload (low) | 3.55 | 0.90 | 39 |
|  |  | Workload (high) | 3.09 | 0.93 | 79 |
|  |  | Total | 3.24 | 0.94 | 118 |

Note: This table contains the original data from 1=best to 5=worst score. In text, the values for “active knowledge transfer” are reversed (that is 5=best score and 1=worst score), because tables and figures are much easier to understand.
