# Supplementary material for: Digital transition in rural emergency medicine: Impact of job satisfaction and workload on communication and technology acceptance
Source: PLoS One. 2023 Jan 24;18(1):e0280956. doi: 10.1371/journal.pone.0280956 (PMC9873191; doi:10.1371/journal.pone.0280956)
Supplement: S1 File — English translation of the German original. (PDF) [file pone.0280956.s004.pdf]

Einwilligung Nutzungsbedingungen und Datenschutz Land|Retter

Hiermit bestätige ich,

_______________________________ (Vorname) _______________________________ (Nachname)
dass ich die Nutzungsbedingungen sowie Datenschutzerklärung zur Teilnahme am Projekt Land|Retter bzw. dem Gesamtsystem corhelp3r gelesen habe und diesen zustimme.

_____________________ _________________________
Ort, Datum Unterschrift

Einwilligung der Verwendung pseudonymisierter Daten Land|Retter

Ich stimme der Verwendung der Daten in pseudonymisierter Form zu.
Ich bestätige durch die Registrierung und Teilnahme als „Land|Retter“, dass ich der Verwendung der pseudonymisierten Einsatzdaten durch den Eigenbetrieb Rettungsdienst Vorpommern-Greifswald, aus Zwecken der internen Qualitätssicherung zustimme. Ich wurde darüber informiert, dass meine Daten dazu unter medizinischen und ökonomischen Gesichtspunkten ausgewertet warden und in anonymisierter Form weitergegeben werden können.

Ich bin auch darüber aufgeklärt worden, dass ich zu jeder Zeit meine Einwilligung ohne negative Konsequenzen zurückziehen kann und die Daten somit nicht für die Qualitätssicherung genutzt werden.

_____________________ _________________________
Ort, Datum Unterschrift

Einwilligung der Verwendung von Emailadresse / Telefonnummer zur Kontaktaufnahme zur wissenschaftlichen Evaluation

Ich stimme der Verwendung meiner hinterlegten Emailadresse sowie Telefonnummer zur Kontaktaufnahme im Rahmen der wissenschaftlichen Evaluation in Form einer Befragung zu.

Bei einer entsprechenden Kontaktaufnahme durch einen Mitarbeiter / Beauftragten des Landkreises oder einer der Evaluationspartner im Projekt Land|Rettung habe ich jederzeit die Möglichkeit, in die geplante Befragung nicht einzuwilligen.

Ich bin auch darüber aufgeklärt worden, dass ich zu jeder Zeit meine Einwilligung ohne negative Konsequenzen zurückziehen kann und die Daten in diesem Fall nicht zur Kontaktaufnahme genutzt werden.

______________________ ______________________

Ort, Datum Unterschrift

English translation of the German original

Project Rural|Rescue

Consent Terms of Use and Data Protection Country| Rescuer

I hereby confirm, ___________________ (Name, Surname) that I comply with the terms of use and privacy policy for participation in the project Rural|Rescue and the Telemedical system and agree to them.

____________________ ________________________

Place, date Signature

Consent to the use of pseudonymised data

I agree to the use of the data in pseudonymised form. I confirm by registering and participating as a "country| Retter", that I agree to the use of the pseudonymised operational data by the in-house company Rettungsdienst Vorpommern-Greifswald, for the purposes of internal quality assurance. I have been informed that my data will be evaluated from a medical and economic point of view and can be passed on in anonymous form .

I have also been informed that I can withdraw my consent at any time without negative consequences and that the data will therefore not be used for quality assurance.

____________________ ________________________

Place, date Signature

Consent to the use of email address / telephone number to contact scientific evaluation

I agree to the use of my stored email address and telephone number to contact you as part of the scientific evaluation in the form of a survey. In the event of a corresponding contact by an employee / representative of the district or one of the evaluation partners in the project Land| Rescue I have the possibility at any time not to consent to the planned survey.

I have also been informed that I can withdraw my consent at any time without negative consequences and that the data will not be used to contact me in this case.

______________________ __________________________

Place, date Signature
