# Supplementary material for: Digital transition in rural emergency medicine: Impact of job satisfaction and workload on communication and technology acceptance
Source: PLoS One. 2023 Jan 24;18(1):e0280956. doi: 10.1371/journal.pone.0280956 (PMC9873191; doi:10.1371/journal.pone.0280956)
Supplement: S2 File — German original, English translation in S1 Appendix. (PDF) [file pone.0280956.s005.pdf]

EvaSys	Land|Rettung Fragebogen zum Telenotarztkonzept	Electric Paper

UNIVERSITÄT GREIFSWALD
Wissen lockt. Seit 1456

Bitte so markieren: ☐ ☒ ☐ ☐ ☐ Bitte verwenden Sie einen Kugelschreiber oder nicht zu starken Filzstift. Dieser Fragebogen wird maschinell erfasst.
Korrektur: ☐ ■ ☐ ☒ ☐ Bitte beachten Sie im Interesse einer optimalen Datenerfassung die links gegebenen Hinweise beim Ausfüllen.

Liebe Teilnehmerin, lieber Teilnehmer, vielen Dank, dass Sie sich die Zeit nehmen, um an unserer Befragung für das Projekt Land|Rettung teilzunehmen. Damit helfen Sie uns, das Telenotarztkonzept weiter zu verbessern.

1. Allgemeiner Part

Zunächst möchten wir Sie um einige Angaben zu Ihrer Person und beruflichen Situation bitten.

1.1 In welcher Art von Organisation arbeiten Sie **im Rahmen der Notfallversorgung** überwiegend?
☐ Leitstelle ☐ Rettungsdienst ☐ Notaufnahme
☐ Verwaltung ☐ Sonstige

1.2 In welcher Position arbeiten Sie aktuell?
☐ (Not-) Arzt/Ärztin ☐ Pflegefachpersonal ☐ Rettungssanitäter
☐ Rettungsassistent ☐ Notfallsanitäter ☐ Disponent
☐ Verwaltung, Management ☐ Sonstige

1.3 Wie lange führen Sie diesen Beruf (unabhängig vom Arbeitgeber) bereits aus?
☐ Unter 2 Jahre ☐ 2-5 Jahre ☐ 6-10 Jahre
☐ 11-20 Jahre ☐ 21-30 Jahre ☐ Über 30 Jahre

1.4 Wie lange sind Sie bereits bei Ihrer derzeitigen Organisation beschäftigt?
☐ Unter 2 Jahre ☐ 2-5 Jahre ☐ 6-10 Jahre
☐ 11-20 Jahre ☐ 21-30 Jahre ☐ Über 30 Jahre

1.5 Haben Sie aktuell eine leitende Position in Ihrer Organisation?
☐ Ja ☐ Nein

1.6 Welches Geschlecht haben Sie?
☐ Männlich ☐ Weiblich ☐ Keine Angabe

2. Notfallmedizin

2.1 Ich fühle mich in der Notfallmedizin kompetent.
Trifft zu ☐ ☐ ☐ ☐ Trifft nicht zu

2.2 Ich erlebe Situationen, in denen ich mir Unterstützung durch einen erfahrenen Kollegen wünsche.
Selten ☐ ☐ ☐ ☐ Häufig

2.3 Dabei wünsche ich mir vor allem Unterstützung bei... (Mehrfachnennung mgl.)
☐ Manuellen Tätigkeiten ☐ Diagnosefindung und Therapieentscheidung ☐ Organisatorischen Tätigkeiten (z.B. Psych-KG)
☐ Ich wünsche mir keine Unterstützung.

2.4 Neben den Angaben in Frage 2.3 wünsche ich mir außerdem Unterstützung bei:

3. Telenotarzt-Konzept

Nun finden Sie einige Aussagen über das Konzept des Telenotarztes.

		Trifft zu	Trifft eher zu	Trifft eher nicht zu	Trifft nicht zu
3.1	Ich halte das Konzept des Telenotarztes für sinnvoll.	☐	☐	☐	☐
3.2	Mir vorgesetzte Personen halten das Konzept des Telenotarztes für sinnvoll.	☐	☐	☐	☐
3.3	Kollegen, die mir wichtig sind, halten das Konzept des Telenotarztes für sinnvoll.	☐	☐	☐	☐
3.4	Personen in meinem Freundeskreis halten das Konzept des Telenotarztes für sinnvoll.	☐	☐	☐	☐
3.5	Wenn ich in einem Projekt wie dem Telenotarzt-Konzept mitmache, steigt mein Ansehen im Kollegenkreis.	☐	☐	☐	☐
3.6	Wenn ich in einem Projekt wie dem Telenotarzt-Konzept mitmache, steigt mein Ansehen im Freundeskreis.	☐	☐	☐	☐
3.7	Ich denke, dass das Konzept des Telenotarztes zu einer relevanten Zeitersparnis führt.	☐	☐	☐	☐
3.8	Ich denke, dass das Konzept des Telenotarztes zu einer schnelleren Diagnosefindung führt.	☐	☐	☐	☐
3.9	Ich denke, dass das Konzept des Telenotarztes zu einem schnelleren Therapiebeginn führt.	☐	☐	☐	☐
3.10	Ich denke, dass das Konzept des Telenotarztes zu einer schnelleren Transportfähigkeit führt.	☐	☐	☐	☐
3.11	Ich denke, dass das Konzept des Telenotarztes meinen Dokumentationsaufwand vermindert.	☐	☐	☐	☐
3.12	Ich denke, dass das Konzept des Telenotarztes meine Arbeitsbelastung vermindert.	☐	☐	☐	☐
3.13	Ich denke, dass das Konzept des Telenotarztes zu einer Verzögerung am Notfallort führt.	☐	☐	☐	☐
3.14	Ich denke, dass das Konzept des Telenotarztes die Qualität der Patientenversorgung verbessert.	☐	☐	☐	☐
3.15	Ich denke, dass das Konzept des Telenotarztes meine berufliche Leistung verbessert.	☐	☐	☐	☐
3.16	Ich denke, dass das Konzept des Telenotarztes meine Effektivität bei der Arbeit steigert.	☐	☐	☐	☐
3.17	Ich denke, dass das Konzept des Telenotarztes nützlich für meine Arbeit ist.	☐	☐	☐	☐
3.18	Ich denke, dass das Konzept des Telenotarztes die etablierte Struktur stört.	☐	☐	☐	☐
3.19	Ich denke, dass der Telenotarzt auch in Situationen alarmiert wird, in denen normalerweise kein Notarzt alarmiert wird.	☐	☐	☐	☐
3.20	Ich arbeite regelmäßig mit dem Telenotarzt zusammen bzw. übernehme regelmäßig Patienten vom Telenotarzt.	☐	☐	☐	☐
3.21	Ich arbeite gern mit dem Telenotarzt zusammen.	☐	☐	☐	☐

EvaSys	Land|Rettung Fragebogen zum Telenotarztkonzept	Electric Paper

3. Telenotarzt-Konzept [Fortsetzung]

		Trifft zu	Trifft eher zu	Trifft eher nicht zu	Trifft nicht zu
3.22	Ich finde es gut, dass das Telenotarzt-Konzept bei uns eingeführt wurde.	☐	☐	☐	☐
3.23	Ich empfehle, dass alle RTW in unserem Landkreis mit der Telenotarzt-Technik ausgestattet werden.	☐	☐	☐	☐
3.24	Ich habe keine Bedenken, den Telenotarzt zu kontaktieren.	☐	☐	☐	☐
3.25	Der Umgang mit modernen Kommunikationsmitteln fällt mir leicht.	☐	☐	☐	☐
3.26	Modernen Kommunikationsmitteln stehe ich privat aufgeschlossen gegenüber.	☐	☐	☐	☐
3.27	Beruflich arbeite ich viel mit modernen Kommunikationsmitteln.	☐	☐	☐	☐
3.28	Ich finde Zusatzfunktionen im Internet, wie z.B. Rote Liste, Giftnotrufzentrale, Leitlinien, hilfreich.	☐	☐	☐	☐
3.29	Ich nutze regelmäßig Zusatzfunktionen im Internet, wie z.B. Rote Liste, Giftnotrufzentrale, Leitlinien.	☐	☐	☐	☐

3.30 Wie häufig nutzen Sie die Telenotarztanwendung?
- ☐ So viel wie möglich
- ☐ Nach keinem festen Schema
- ☐ Nie
- ☐ Immer, wenn sie mich bei einer Aufgabe unterstützen kann

3.31 Haben Sie außerhalb des Projektes Land|Rettung bereits bei telemedizinischen Projekten mitgemacht? Ja ☐ ☐ Nein

3.32 Kannten Sie die Telenotarztanwendung bereits vor der heutigen Befragung? Ja ☐ ☐ Nein

3.33 Welche Vorteile sehen Sie durch die Einführung der Telenotarztanwendung?

3.34 Vermutlich hatten Sie vor der Einführung der Telenotarztanwendung bestimmte Erwartungen. Welche haben sich erfüllt und welche nicht?

MUSTER

4. Wissensarten

Im folgenden Abschnitt geht es nun darum, welche Arten von Wissen in der Organisation, in der Sie beschäftigt sind, vorrangig gefördert und angewendet werden.

		Stimme voll zu	Stimme zu	Stimme eher zu	Stimme eher nicht zu	Stimme nicht zu	Stimme überhaupt nicht zu
4.1	Die Einarbeitung in die Nutzung von neuen Arbeitsmitteln, neuer Software, etc. geschieht meistens durch persönlichen Austausch und Beobachtung am Arbeitsplatz.	☐	☐	☐	☐	☐	☐
4.2	Für die Weiterentwicklung aller Mitarbeiter gibt es in unserer Organisation regelmäßige Mitarbeitergespräche oder entsprechende Berichts- oder Fragebogen.	☐	☐	☐	☐	☐	☐
4.3	In unserer Organisation finden regelmäßig team- bzw. berufsgruppenübergreifende Treffen statt, um die Zusammenarbeit untereinander zu verbessern.	☐	☐	☐	☐	☐	☐
4.4	Die Einhaltung von Verfahrensanweisungen, sowie der Ablauf von Prozessen wird in unserer Organisation umfassend überprüft und ständig verbessert (z.B. Dokumentation).	☐	☐	☐	☐	☐	☐
4.5	Die Abläufe in unserer Organisation (z.B. Notrufannahme, Schockraumübergabe, Fahrzeugdesinfektion) sind genau festgelegt und jedem Mitarbeiter soweit nötig bekannt.	☐	☐	☐	☐	☐	☐
4.6	Bei wichtigen Entscheidungen kann jeder Mitarbeiter seine eigenen Argumente einbringen und wird in den Entscheidungsprozess einbezogen.	☐	☐	☐	☐	☐	☐
4.7	Die Mitarbeiter unserer Organisation gehen auch außerhalb der Arbeitszeiten gemeinsamen Aktivitäten nach (z.B. sportlich oder kulturell).	☐	☐	☐	☐	☐	☐
4.8	In unserer Organisation stimmen sich auch kurzfristig zusammengestellte Teams und Schichtgruppen schnell ab und arbeiten dann produktiv und reibungslos zusammen.	☐	☐	☐	☐	☐	☐
4.9	Wenn man etwas schlecht mit Worten erklären kann, sondern vormachen muss (z.B. Bedienung neuer Geräte), findet sich immer ein Kollege, der das übernimmt.	☐	☐	☐	☐	☐	☐
4.10	In unserer Organisation achten die Mitarbeiter darauf, dass jeder seine Arbeit regelkonform ausführt und weisen Kollegen ggf. direkt auf Fehlverhalten hin.	☐	☐	☐	☐	☐	☐
4.11	In unserem Team / Bereich kennen wir uns sehr gut und wissen unsere Stärken und Schwächen so einzusetzen und zu kompensieren, dass wir uns gegenseitig ideal ergänzen.	☐	☐	☐	☐	☐	☐
4.12	In unserem Team / Bereich ist klar geregelt, wer für welche Themenbereiche verantwortlich und Ansprechpartner/in für alle anderen ist.	☐	☐	☐	☐	☐	☐

MUSTER

5. Wissensquellen

In diesem Abschnitt geht es darum zu erfahren, welche Methoden und Medien in Ihrer Organisation vorrangig genutzt werden, um Informationen und Wissen auszutauschen.

		Stimme voll zu	Stimme zu	Stimme eher zu	Stimme eher nicht zu	Stimme nicht zu	Stimme überhaupt nicht zu
5.1	In unserer Organisation stehen ausreichend Dokumente zu Fragen des Arbeitsalltags sowie relevante Fachliteratur zur Verfügung (z.B. SOP, Fachzeitschriften).	☐	☐	☐	☐	☐	☐
5.2	Arbeitsergebnisse, Prozessveränderungen, etc. werden dokumentiert (z.B. Therapieerfolgsrate, Umstellung auf neue Softwareanwendungen -> „lessons learned“).	☐	☐	☐	☐	☐	☐
5.3	Unsere Organisation wird von externen Experten unterstützt (z.B. Mitarbeiter von Medizinprodukte-Herstellern, Software-Anbietern).	☐	☐	☐	☐	☐	☐
5.4	Es gibt einen regelmäßigen Informationsaustausch mit anderen Organisationen der Rettungs- bzw. Behandlungskette, die vor oder nach unserer Organisation Teil der Patientenversorgung sind (z.B. Leitstelle, Praxis, Rettungsdienst, Klinik).	☐	☐	☐	☐	☐	☐
5.5	Es gibt interne Qualitätszirkel zu internen Neuerungen und Ideenmanagement, die in unserer Organisation der ständigen Verbesserung und Qualitätssicherung dienen.	☐	☐	☐	☐	☐	☐
5.6	Unsere Organisation arbeitet erfolgreich mit externen Einrichtungen (z.B. Hochschulen, Instituten) in der Forschung und Entwicklung von Behandlungen, einsatztaktischen Strategien, etc. zusammen.	☐	☐	☐	☐	☐	☐
5.7	Unsere Organisation nutzt regelmäßig externe Fort- und Weiterbildungsangebote (z.B. Konferenzen, Kongresse, Seminare) in der Region.	☐	☐	☐	☐	☐	☐
5.8	Öffentliche Forschungseinrichtungen oder zuständige Institutionen und Träger liefern unserer Organisation regelmäßig praxisrelevante Forschungs- bzw. Studienergebnisse.	☐	☐	☐	☐	☐	☐
5.9	Neue Mitarbeiter erhalten in unserer Organisation Beratung und Einarbeitungsunterstützung zu ihrer Arbeitsstelle.	☐	☐	☐	☐	☐	☐
5.10	Übergeordnete Institutionen (z.B. Träger, Zweckverbände) unterstützen unsere Organisation aktiv bei der fachlichen Entwicklung und Problemlösung.	☐	☐	☐	☐	☐	☐
5.11	In unserer Organisation werden interne Seminare, Workshops, etc. angeboten, die von internen Experten des Hauses geleitet werden.	☐	☐	☐	☐	☐	☐
5.12	In unserer Organisation werden interne Seminare, Workshops, etc. angeboten, die von externen Experten anderer Unternehmen oder Institutionen geleitet werden.	☐	☐	☐	☐	☐	☐
5.13	Es gibt ausreichend Angebote für den Wissensaustausch zwischen Organisationen, die gemeinsame Interessen haben (z.B. interdisziplinäre Trainings, Fallkonferenzen).	☐	☐	☐	☐	☐	☐
5.14	Unsere Organisation erhält regelmäßig Informationen von externen Firmen oder Institutionen (z.B. Medizinprodukte-Hersteller) zu deren Produkten und Dienstleistungen und wird von diesen auch bei der fachlichen Entwicklung unterstützt.	☐	☐	☐	☐	☐	☐

MUSTER

6. Wissensinstrumente

Im nächsten Abschnitt möchten wir erfahren, welche Methoden und Medien in Ihrer Organisation vorrangig genutzt werden, um Informationen und Wissen auszutauschen.

6.1 Gibt es in Ihrer Organisation eine Sammlung von internen SOP (Standard Operating Procedures) bzw. Verfahrensanweisungen für bestimmte Vorgänge und Situationen?

☐ Ja ☐ Nein --> dann bitte weiter mit Frage 6.11

Antwortskala: Stimme voll zu / Stimme zu / Stimme eher zu / Stimme eher nicht zu / Stimme nicht zu / Stimme überhaupt nicht zu

Nr.	Aussage	Antwort
6.2	In den verfügbaren Dokumenten (digital oder analog) finde ich schnell und genau die Informationen, nach denen ich gesucht habe.	☐ ☐ ☐ ☐ ☐ ☐
6.3	Die Dokumente sind für alle Mitarbeiter verfügbar und repräsentieren keine Meinungen von Einzelpersonen, sondern zeigen allgemein gültige und anerkannte Vorgehensweisen bzw. einheitliche Standards unserer Organisation auf.	☐ ☐ ☐ ☐ ☐ ☐
6.4	Sämtliche Dokumente sind übersichtlich und transparent abgelegt, sodass ich schnell erkennen kann, welches der verfügbaren Dokumente mir bei einem bestimmten Problem oder einer bestimmten Aufgabe weiterhelfen wird.	☐ ☐ ☐ ☐ ☐
6.5	An meinem Arbeitsplatz kann ich stets auf die entsprechenden Dokumente zugreifen, da ausreichend Ordner (analog) bzw. PCs etc. (digital) vorhanden sind.	☐ ☐ ☐ ☐ ☐
6.6	Die entsprechenden Dokumente sind immer leicht zu finden und man kann schnell und unkompliziert auf sie zugreifen.	☐ ☐ ☐ ☐ ☐
6.7	Die verfügbaren Dokumente sind stets auf einem aktuellen Stand und werden umgehend an veränderte Bedingungen angepasst.	☐ ☐ ☐ ☐ ☐
6.8	Die enthaltenen Informationen bzw. Anweisungen helfen mir systematisch und genau, eine konkrete Problem- oder Aufgabenstellung zu bearbeiten.	☐ ☐ ☐ ☐ ☐
6.9	Ich entscheide selbst, wann ich entsprechende Vorgaben aufrufe und diese ein- bzw. umsetze.	☐ ☐ ☐ ☐ ☐
6.10	Durch die Nutzung der SOP und Verfahrensanweisungen verbessert sich meine eigene Kompetenz zur Bearbeitung verschiedener Aufgaben wesentlich.	☐ ☐ ☐ ☐ ☐

MUSTER

6. Wissensinstrumente [Fortsetzung]

6.11 Gibt es im regionalen Umfeld Ihrer Organisation externe Netzwerke oder Arbeitskreise, von denen Sie Informationen erhalten bzw. denen Sie beitreten können? (z.B. regionale Arbeitskreise, themenbezogene Projektgruppen, Fachgesellschaften)

☐ Ja ☐ Nein --> dann bitte weiter mit Frage 7.1

		Stimme voll zu	Stimme zu	Stimme eher zu	Stimme eher nicht zu	Stimme nicht zu	Stimme überhaupt nicht zu
6.12	In solchen Informationsangeboten finde ich schnell genau die Informationen, nach denen ich gesucht habe.	☐	☐	☐	☐	☐	☐
6.13	Die Informationsanbieter nutzen unabhängig vom entsendenden Arbeitgeber die Kompetenzen aller Mitglieder, um das gemeinsame Thema voranzubringen.	☐	☐	☐	☐	☐	
6.14	In solchen Informationsangeboten wird mir sehr schnell deutlich, wer mir welche Information liefern könnte oder wer welche Kompetenzen besitzt, die mir bei meinen eigenen Problem- bzw. Aufgabenstellungen hilfreich sein könnten.	☐	☐	☐	☐	☐	
6.15	Möchte ich selbst zu einem Informationsangebot beitragen, ist dies aus meiner Sicht unkompliziert und schnell möglich, da ausreichend Möglichkeiten bestehen.	☐	☐	☐	☐	☐	
6.16	Die Teilnahme an solchen Informationsangebote, d.h. Beitritt, Mitautorenschaft, etc., geschieht aus meiner Sicht immer unkompliziert und ohne großen Zeitverzug.	☐	☐	☐	☐	☐	
6.17	Derartige Informationsangebote befassen sich stets mit aktuellen Themen und Entwicklungen und passen ihre Ausrichtung diesen fortlaufend an.	☐	☐	☐	☐	☐	
6.18	Informationen und Kontakte aus entsprechenden Informationsangeboten helfen mir gezielt, Lösungen für Problem- und Aufgabenstellungen meiner Arbeit zu finden.	☐	☐	☐	☐	☐	
6.19	Ich entscheide selbst, ob und in welchem zeitlichen Umfang ich bei entsprechenden Informationsangeboten aktiv werde.	☐	☐	☐	☐	☐	
6.20	Die Teilnahme an Informationsangeboten verbessert wesentlich meine Kompetenzen zur Lösung von Problemen und Aufgaben im Arbeitsalltag.	☐	☐	☐	☐	☐	

MUSTER

7. Arbeitszufriedenheit

*Bitte bewerten Sie nun folgende Aussagen zu Ihrem Arbeitsalltag, indem Sie Ihr persönliches Empfinden auf der Skala von 1 bis 10 einordnen. Bitte setzen Sie pro Frage nur **ein Kreuz** (1= linke Aussage trifft voll zu, 5 = weder linke noch rechte Aussage trifft zu, 10= rechte Aussage trifft voll zu).*

Nr.	Linke Aussage	1	2	3	4	5	6	7	8	9	10	Rechte Aussage
7.1	Für meine Arbeit bekomme ich ausreichend Wertschätzung und Unterstützung von meinen Kollegen.	☐	☐	☐	☐	☐	☐	☐	☐	☐	☐	Meine Arbeit wird von meinen Kollegen wenig wertgeschätzt und unnötig kritisiert.
7.2	Für meine Arbeit bekomme ich ausreichend Wertschätzung und Unterstützung von meinen Vorgesetzten.	☐	☐	☐	☐	☐	☐	☐	☐	☐	☐	Meine Arbeit wird von meinen Vorgesetzten wenig wertgeschätzt und unnötig kritisiert.
7.3	Meine Vorgesetzten kennen meine persönlichen Ziele und berücksichtigen diese, soweit möglich.	☐	☐	☐	☐	☐	☐	☐	☐	☐	☐	Meine persönlichen Ziele werden durch meine Vorgesetzten weder wahrgenommen noch berücksichtigt.
7.4	Auf Zusagen, die mein Arbeitgeber und meine Vorgesetzten gemacht haben, kann man sich stets verlassen.	☐	☐	☐	☐	☐	☐	☐	☐	☐	☐	Zusagen kann man nicht trauen, weil sie nicht eingehalten werden.
7.5	Wann und wo ich arbeiten muss, kann ich vorausschauend planen.	☐	☐	☐	☐	☐	☐	☐	☐	☐	☐	Arbeitsorte und -Zeiten sich für mich nicht planbar.
7.6	Über Planungen und Entscheidungen, die meine Arbeit betreffen, werde ich rechtzeitig und ausreichend informiert.	☐	☐	☐	☐	☐	☐	☐	☐	☐	☐	Über für mich wichtige Planungen und Entscheidungen werde ich nicht rechtzeitig und ausreichend informiert.
7.7	Entscheidungen, die meine Arbeit betreffen sowie der Entscheidungsprozess, sind für mich gut nachvollziehbar.	☐	☐	☐	☐	☐	☐	☐	☐	☐	☐	Entscheidungen und Entscheidungsprozesse, die mich betreffen, kann ich nicht nachvollziehen.
7.8	Im vorgegebenen Rahmen kann ich selbst bestimmen, wie ich meine Arbeit mache.	☐	☐	☐	☐	☐	☐	☐	☐	☐	☐	Ich habe keinen Entscheidungsspielraum und fühle mich in meiner Arbeit durch Vorgaben gegängelt.
7.9	Die Arbeit bietet viele Herausforderungen, ich fühle mich dabei aber nie überfordert.	☐	☐	☐	☐	☐	☐	☐	☐	☐	☐	Durch die Anforderungen meiner Arbeit fühle ich mich überfordert.
7.10	Im Rahmen meiner Beschäftigung in der Notfallversorgung mache ich eine sinnvolle Arbeit, die der Gesellschaft nützt.	☐	☐	☐	☐	☐	☐	☐	☐	☐	☐	Ich verrichte sinnlose Arbeiten, die niemandem nützen.
7.11	Nur bei meinem aktuellen Arbeitgeber finde ich die Arbeitsbedingungen, die mir wichtig sind und die ich haben möchte.	☐	☐	☐	☐	☐	☐	☐	☐	☐	☐	Ich könnte genauso gut bei einem anderen Arbeitgeber in der Notfallversorgung arbeiten.
7.12	Für meine Arbeit im Rahmen der Notfallversorgung werde ich gerecht und angemessen bezahlt.	☐	☐	☐	☐	☐	☐	☐	☐	☐	☐	Für die Arbeit, dich ich im Rahmen der Notfallversorgung leiste, werde ich nicht ausreichend bezahlt.

7. Arbeitszufriedenheit [Fortsetzung]

		1	2	3	4	5	6	7	8	9	10	
7.13	Meine derzeitige Position in der Notfallversorgung bietet mir optimale Möglichkeiten, mich zu entwickeln und in meinem Beruf Karriere zu machen.	☐	☐	☐	☐	☐	☐	☐	☐	☐	☐	Meine derzeitige Position in der Notfallversorgung sehe ich als Sackgasse, in der ich mich beruflich nicht entwickeln kann.
7.14	Nach meiner Erfahrung ist die Arbeitsbelastung bei meinem Arbeitgeber in der Notfallversorgung nicht zu hoch und das wird auch so bleiben.	☐	☐	☐	☐	☐	☐	☐	☐	☐	☐	Nach meiner Erfahrung ist die Arbeitsbelastung unerträglich und es wird in Zukunft nicht besser.

7.15 Für wie wahrscheinlich halten Sie es, dass Sie in fünf Jahren noch bei Ihrem aktuellen Arbeitgeber (Notfallversorgung) tätig sein werden? **(Achtung: Skala beachten!)**

Sehr unwahrscheinlich ☐ ☐ ☐ ☐ ☐ ☐ ☐ ☐ ☐ ☐ Äußerst wahrscheinlich

7.16 Läuft Ihr Arbeitsvertrag in weniger als fünf Jahren aus? Ja ☐ ☐ Nein

7.17 Gibt es sonst noch Dinge, die Sie uns gerne mitteilen möchten?

Vielen Dank für die Beantwortung der Fragen.

Land | Rettung

Landkreis Vorpommern-Greifswald · UNIVERSITÄT GREIFSWALD Wissen lockt. Seit 1456 · Universitätsmedizin GREIFSWALD · zeb business school der Steinbeis-Hochschule Berlin
