# Supplementary material for: Digital transition in rural emergency medicine: Impact of job satisfaction and workload on communication and technology acceptance
Source: PLoS One. 2023 Jan 24;18(1):e0280956. doi: 10.1371/journal.pone.0280956 (PMC9873191; doi:10.1371/journal.pone.0280956)
Supplement: Attachment — Submitted filename: resonse to reviewer PLOS ONE.docx [file pone.0280956.s007.docx]

**Response to the editors and reviewers**

**Response to the editors**

(response to the reviewers: see below)

*1. Please ensure that your manuscript meets PLOS ONE's style requirements, including those for file naming. The PLOS ONE style templates …*

- We reformatted the manuscript according to the formatting samples (…body and …title_authors_affiliation). The references were re-formatted according to PLOS ONE / Chicago style.

*2. Please provide additional details regarding participant consent. In the ethics statement in the Methods and online submission information, please ensure that you have specified what type you obtained (for instance, written or verbal, and if verbal, how it was documented and witnessed). If your study included minors, state whether you obtained consent from parents or guardians. If the need for consent was waived by the ethics committee, please include this information.*

- Information about the consent (written, informed consent) has been added to the Method section and a supplementary file with the consent form (German original and English translation). No minors were included in this study.

*3. Please update your submission to use the PLOS LaTeX template. The template and more information on our requirements for LaTeX submissions can be found at* [*http://journals.plos.org/plosone/s/latex*](http://journals.plos.org/plosone/s/latex)*.*

- The manuscript has been prepared in the MS Word “docx” format, the beforementioned format samples have been used.

*4. Thank you for stating the following in the Acknowledgments Section of your manuscript:*

“This research has been funded by a €5.38 million grant from Germany’s Innovation Fund (01NVF16004). Ethical approval BB 044/17 has been granted from Ethics Commission University Medicine Greifswald.”

*We note that you have provided funding information that is not currently declared in your Funding Statement. However, funding information should not appear in the Acknowledgments section or other areas of your manuscript. We will only publish funding information present in the Funding Statement section of the online submission form.*

*Please remove any funding-related text from the manuscript and let us know how you would like to update your Funding Statement. Currently, your Funding Statement reads as follows:*

“All authors participated in the funded project.

This research has been funded by a €5.38 million grant from Germany’s Innovation Fund (Innovationsfonds des Gemeinsamen Bundesausschusses, G-BA) under number 01NVF16004.

URL: <https://innovationsfonds.g-ba.de/projekte/neue-versorgungsformen/landrettung-zukunftsfeste-notfallmedizinische-neuausrichtung-eines-landkreis.63>

The funding institution had no role in study design, data collection and analysis, decision to publish, or preparation of the manuscript.”

*Please include your amended statements within your cover letter; we will change the online submission form on your behalf.*

- We have added a corrected funding statement to the revised version of the cover letter. Thank you very much for supporting us!

*5. Thank you for stating the following in your Competing Interests section:*

“All authors declare no competing interests.”

*Please complete your Competing Interests on the online submission form to state any Competing Interests. If you have no competing interests, please state ""The authors have declared that no competing interests exist."", as detailed online in our guide for authors at* [*http://journals.plos.org/plosone/s/submit-now*](http://journals.plos.org/plosone/s/submit-now)

*This information should be included in your cover letter; we will change the online submission form on your behalf.*

- We have added a statement concerning competing interests to the revised version of the cover letter. Again, thank you very much for supporting us!

*6. In your Data Availability statement, you have not specified where the minimal data set underlying the results described in your manuscript can be found. PLOS defines a study's minimal data set as the underlying data used to reach the conclusions drawn in the manuscript and any additional data required to replicate the reported study findings in their entirety. All PLOS journals require that the minimal data set be made fully available. For more information about our data policy, please see* [*http://journals.plos.org/plosone/s/data-availability*](http://journals.plos.org/plosone/s/data-availability)*.*

*Upon re-submitting your revised manuscript, please upload your study’s minimal underlying data set as either Supporting Information files or to a stable, public repository and include the relevant URLs, DOIs, or accession numbers within your revised cover letter. For a list of acceptable repositories, please see* [*http://journals.plos.org/plosone/s/data-availability#loc-recommended-repositories*](http://journals.plos.org/plosone/s/data-availability#loc-recommended-repositories)*. Any potentially identifying patient information must be fully anonymized.*

*Important: If there are ethical or legal restrictions to sharing your data publicly, please explain these restrictions in detail. Please see our guidelines for more information on what we consider unacceptable restrictions to publicly sharing data:* [*http://journals.plos.org/plosone/s/data-availability#loc-unacceptable-data-access-restrictions*](http://journals.plos.org/plosone/s/data-availability#loc-unacceptable-data-access-restrictions)*. Note that it is not acceptable for the authors to be the sole named individuals responsible for ensuring data access.*

*We will update your Data Availability statement to reflect the information you provide in your cover letter.*

- The full raw data set has been uploaded as supplementary file (in SPSS format). The data availability has been stated in the cover letter and has been listed as supplementary file in the manuscript.

*7. Your ethics statement should only appear in the Methods section of your manuscript. If your ethics statement is written in any section besides the Methods, please delete it from any other section.*

- The ethic statement has been amended and moved to the Method section.

**Response to the reviewers**

(response to the editors: see above)

We would like to thank the anonymous reviewers for their helpful comments and recommendations helping us to improve our manuscript!

All changes in the manuscript (tracked version) have been highlighted in yellow. In what follows, we respond to the specific comments of the reviewer, individually. We have highlighted the aspects of the reviewers comments we are referring to in our response.

*Reviewers’ comments are shown in italics.*

- Our response is marked with an 🡪

*This is a potentially interesting study and the author(s) tried to address this research issue in rural emergency medicine. Specifically, the title “Digital transition in rural emergency medicine: Impact of job satisfaction and workload on communication and technology acceptance”.*

*However, this manuscript does satisfy the rigor and precision required for the work. I think the most important reason is that this paper clarity in theoretical support in Introduction section and hereafter in the literature review as well as implications all look good and properly well-designed study. Some comments and suggestions for improvement are as follows.*

*In the abstract section: this sentence can be revised “Data were collected via machine readable questionnaires issued to 755 persons (411 pre, 344 post), of which 304 or 40.3% of these persons responded (194 pre, 115 post)”.*

- We have added this sentence in the abstract and adapted the abstract in order to make purpose and implications of the study clearer.

*At page 7, in design section, “During the first phase of the study (pre), 411 questionnaires were issued over a period of three months, of which 194 completed and evaluable questionnaires were returned (response rate 47.2%).” Is there any proper reason of lower response? Although, the response rate for any given survey may be one of the least important factors in analysing your results. There is no "accepted" response rate. It is accurate to say the higher the better, though. But author(s) needs to provide the proper reason and justification on this issue. Telephone surveys historically had the highest response rates, but they were also the most expensive. Old fashioned snail mail surveys had the lowest response rates, but they were cheaper. Technology continues to change survey research methods. Moreover, your response rate may differ depending on your population and geographic area. So researcher goal is not a high response rate, but the representation of the population of interest and low non-response bias (your entire data collection instrument affects this: population of interest, sampling, survey type, questions, benefit for the respondent, etc). But in your case situation is entirely different. So, you need to provide justification from your context. I hope you understand the importance of response rate in study. Sometimes researcher easily ignore the importance of this. So don’t be.*

- We added an explanation about the response rate as well as a comparison to the response rates found in similar studies. We also discuss the issue of a possible non-response bias. We added an explanation why the response rate in the post-phase of the study is lower than in the pre-phase.

*One last thing. For example: “Based on extensive preliminary studies published elsewhere [54-56], we combined these aspects in a polarity profile, which includes questions to be scored from 1 (positive pole) to 10 (negative pole; see table 2). An example question concerning support from colleagues reads as follows: "For my work I get sufficient appreciation and support from my colleagues" (positive pole=1) or "My work is not appreciated and unnecessarily criticized by my colleagues" (negative pole=10). The scale is highly reliable with Cronbach’s Alpha = 0.91 in this and 0.78 in prior studies [56].” Based on the preliminary studies published elsewhere? Is there any justification from the literature?*

- We added the exact references and clarified which reliability was found in what study.

*According to my point of view, it is not necessary you combine the variables into the same or similar scale since they are measuring different constructs. Once they have been appropriately sectionalized and the items in the different sections have been content validated, it's okay.*

- We calculated and stated reliability (Cronbach’s Alpha) of each section of the survey we used as a factor in the analyses in text.

*However, the reliably coefficient of each of the sections in line with the various variables under investigation should be separately calculated and results of the data collected from the use of the questionnaire should be reported separately in line with the variables and research objectives. I believe the author(s) should first address these fundamental flaws.*

- We arranged the order, in which the results are presented and added 3 tables reporting the results in line with the hypotheses and the analyses testing them. We moved an additional table (breakdown of means) to appendix 3.
