# Supplementary material for: Digital transition in rural emergency medicine: Impact of job satisfaction and workload on communication and technology acceptance
Source: PLoS One. 2023 Jan 24;18(1):e0280956. doi: 10.1371/journal.pone.0280956 (PMC9873191; doi:10.1371/journal.pone.0280956)
Supplement: Attachment — Submitted filename: Response to Reviewers.docx [file pone.0280956.s008.docx]

PONE-D-22-04801R1

Digital transition in rural emergency medicine: Impact of job satisfaction and workload on communication and technology acceptance

**Rebuttal Letter**

Dear reviewers, dear editors, dear Dr. Rafiq,

we are grateful for your helpful comments and the opportunity to improve our manuscript. In what follows, we go through your requirements, step by step and in line with the structure of the manuscript (that is, from “introduction” to “discussion”).

Sincerely,

Joachim Hasebrook (in the name of all authors)

______________________________________

- The introduction section should depict the need for this study and must establish the rational of grouping these variables

*We added a paragraph about the need for this study, and why job satisfaction, workload and communication are essential. We also added theoretical background about these aspects and their interrelation.*

- The authors should also provide details regarding the theories used to underpin the relationship

*We added details about the theories underpinning the different factors and their relationship.*

- There is a need for theoretical model of this study
*We added a section about the “Research model and Hypotheses” explaining the theoretical model behind our research.*

- The authors should highlight the hypotheses in a way that those should correspond to the literature.

*In the same section, we derived our hypothesis from the theoretical model and explicitly stated which references are related to the different hypotheses.*

- in-depth addressing for the validity issues

*We added paragraphs in the section “Material” about each instrument (job satisfaction and demands, technology acceptance, knowledge sharing) with details not only about reliability but also validity of the instruments applied in our study.*

- how the researcher/s get the results

*We added a paragraph in the section “Procedure” to explain how the results were gathered with machine-readable surveys, on site.*

- explain the position of the results of the study to previous studies

*We added text passages and paragraphs in the section “Discussion” to lay out, where our results support and contradict previous studies.*

- give a detail discussion on the future research directions. Specifically, the research has not explained the implications of the research

*We added paragraphs to both sections, “Future Research” and “Practical Implications”, to explain future research directions and their implications.*

- The authors should proofread the manuscript once again for any potential errors.

*We asked a professional proofreading service to improve our manuscript.*

- We recommend that you deposit your laboratory protocols in protocols.io

*The complete data set has been uploaded as supplementary file (S3)*

**Thanks very much to the editor and the reviewer for the opportunity
to revise and improve our manuscript!**
